# Patient Characteristics and Outcomes of Radial to Femoral Access-Site Crossover

**DOI:** 10.1016/j.jscai.2024.102450

**Published:** 2025-01-21

**Authors:** Revathy Sampath-Kumar, Ehtisham Mahmud, Kerem Korkmaz, Lawrence Ang, Belal Al Khiami, Anna Melendez, Ryan Reeves, Ori Ben-Yehuda

**Affiliations:** UCSD Division of Cardiovascular Medicine, Sulpizio Cardiovascular Center, La Jolla, California

**Keywords:** access site, crossover, percutaneous coronary intervention, transfemoral, transradial

## Abstract

**Background:**

The need for radial to femoral access-site crossover (RFC) remains a limitation of radial percutaneous coronary intervention (PCI) with unknown implications.

**Methods:**

The UC San Diego Health internal National Cardiovascular Data Registry CathPCI Registry was used to obtain data on patients who underwent PCI from January 2018 to September 2022 for any indication. Coronary artery bypass graft patients were excluded. Patient- and procedure-level predictors of RFC, complications, and all-cause mortality within 1-year post-PCI were assessed.

**Results:**

A total of 3054 patients were included with a mean age of 67 ± 12 years, and 43.2% had acute coronary syndrome. Of these patients, 109 required RFC, 2287 had successful radial access (RA), and 658 had successful femoral access. There were no differences in comorbidities between the RFC and RA groups. Patients who required RFC had 29% longer fluoroscopy time and 16% more contrast volume compared to patients who had RA. Independent predictors of RFC were age >70 years (OR, 2.68; 95% CI, 1.79-4.01; *P* < .001), vasopressor support at the time of PCI (OR, 2.87; 95% CI 1.33-6.20; *P* = .007), and dialysis dependence (OR, 3.05; 95% CI, 1.34-6.93; *P* = .008). Patients who required RFC had higher 30-day all-cause mortality (3.7% vs 1.0%, *P* = .028), bleeding complications (8.3% vs 2.6%, *P* = .003), and need for blood products (7.3% vs 1.4%, *P* < .001) compared to patients who had RA. There was no difference in all-cause mortality or complications between the RFC and femoral access groups.

**Conclusions:**

Radial to femoral access-site crossover was associated with higher short-term mortality and bleeding complications compared to RA. Age greater than 70 years, vasopressor support, and dialysis dependence were associated with RFC.

## Introduction

Optimal access-site selection is critically important to ensure procedural success and minimize complications during percutaneous coronary intervention (PCI). Radial access (RA) has been associated with lower bleeding complications, vascular complications, mortality, and cost compared to femoral access (FA).^1^, ^2^, ^3^, ^4^, ^5^, ^6^ These benefits are amplified in the setting of acute coronary syndromes (ACS) and in high-volume radial centers.^3^ Therefore, societal guidelines recommend an initial radial approach when technically feasible.^7^^,^^8^ The need for radial to femoral access-site crossover (RFC) remains a limitation of radial PCI with unknown implications. Rates of failed RA requiring RFC range from 3% to 10% in randomized trials.^9^ Feasibility of RA may be unclear prior to the procedure.

Studies have identified variables that may predict the need for RFC including female sex, age, height, operator experience, cardiogenic shock, and certain comorbidities but results have not been consistent.^10^, ^11^, ^12^, ^13^, ^14^, ^15^, ^16^, ^17^, ^18^ Most of these studies were conducted when there was limited operator experience with RA before the use of ultrasound guidance, and only a few included non-ACS patients. This variability underscores the need to elucidate the underlying mechanisms of RFC in order to develop strategies to minimize its occurrence, particularly in high-risk populations where RFC may have significant clinical implications.

RFC has been associated with access-site complications, bleeding, and longer procedural time compared to RA.^10^^,^^12^^,^^13^^,^^19^ Yet in ST-segment elevation myocardial infarction (STEMI), RFC has not been associated with higher door-to-reperfusion time.^20^ There are limited studies assessing the impact of RFC on mortality, with conflicting results.^10^^,^^12^^,^^15^^,^^19^^,^^20^ Some studies found higher in-hospital and long-term mortality in STEMI patients who required RFC compared to those who had RA,^10^^,^^15^^,^^20^ whereas other studies found no difference.^12^^,^^19^ Studies have not evaluated mortality associated with RFC in non-ACS patients.

We thus aimed to assess the mechanism and rate of RFC along with the impact of RFC on outcomes including mortality and complications among a large contemporary cohort of patients who underwent PCI for any indication at a high-volume radial center.

## Methods

### Data source

The UC San Diego Health internal National Cardiovascular Data Registry (NCDR) CathPCI Registry was used to obtain data on patients who underwent PCI from January 2018 to September 2022 at UC San Diego Health sites in La Jolla or Hillcrest. Data were abstracted by a dedicated nurse in the Cardiovascular Outcomes Group at UC San Diego Health. UC San Diego is an academic tertiary and quaternary referral hospital system that is a high-volume radial center. PCI procedures were performed by attending interventional cardiologists with or without interventional cardiology fellows.

RFC events and bleeding complications recorded in the database were adjudicated using the electronic medical record. Reasons for RFC were obtained from the PCI procedure log and review of angiography films. All-cause mortality up to 1-year post-PCI was obtained from the electronic medical record confirmed by the California Department of Public Health vital records and decedent records maintained by the Health Information Management team at UC San Diego. The Institutional Review Board of the University of California, San Diego approved the study (#809443).

### Patient selection

**Figure 1:**
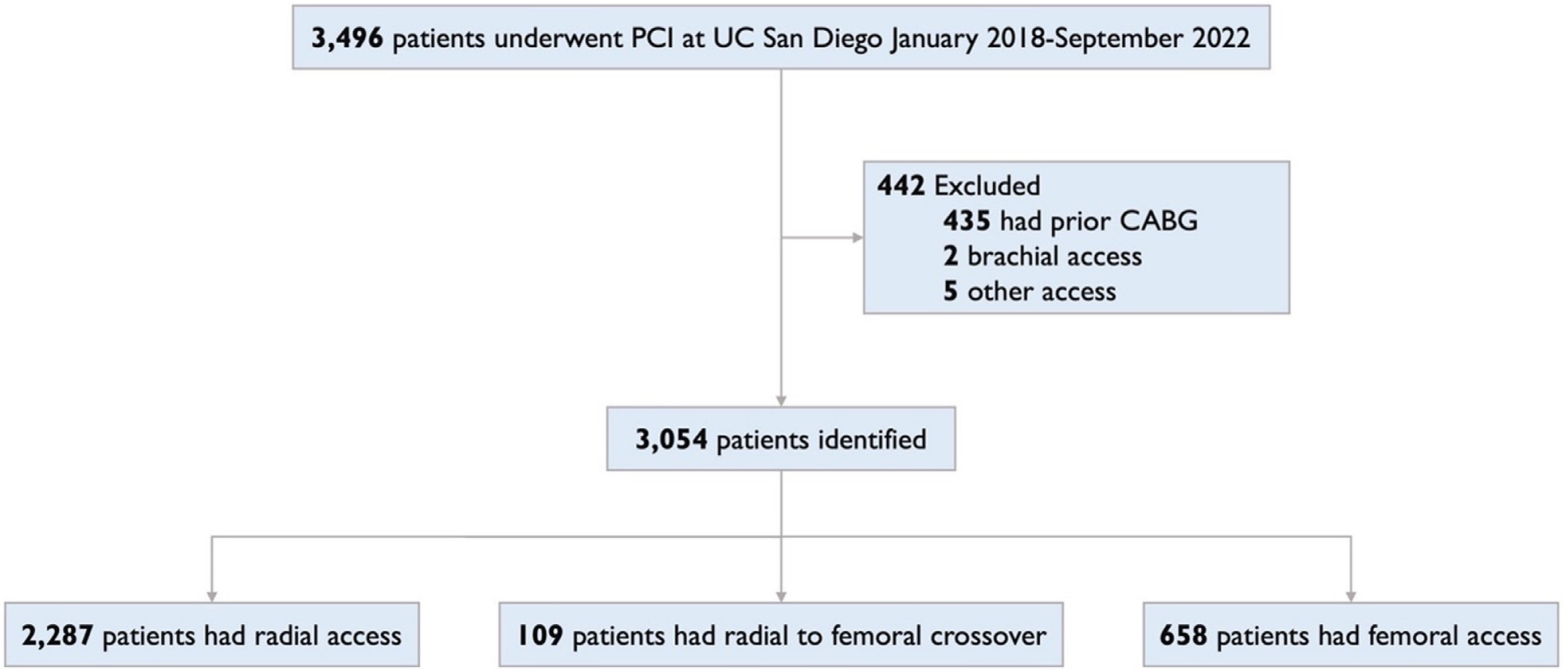
**Consolidated Standards of Reporting Trials (CONSORT) diagram of patients included in the study.** CABG, coronary artery bypass graft; PCI, percutaneous coronary intervention;

A total of 3496 adult patients underwent PCI at UC San Diego Health from January 2018 to September 2022. Among these patients, 435 patients had coronary artery bypass grafts and were excluded. Patients undergoing PCI with brachial or alternative access (n = 7) were also excluded. The final cohort had 3054 patients of whom 109 required RFC, 2287 had RA, and 658 had FA (Figure 1). Ultrasound guidance was used for all FA cases. Ultrasound guidance was not routinely used for RA cases which were all right radial. Our institutional practice for RA failure is to crossover to FA. There were no cases of crossover to alternative forearm access. This study followed the Strengthening the Reporting of Observational Studies in Epidemiology reporting guidelines.

### End points and definitions

Outcomes assessed were ischemic major adverse cardiovascular events (MACE) and bleeding complications that occurred between the procedure and hospital discharge, and all-cause mortality up to 1-year post-PCI. Definitions for PCI-related myocardial infarction, stroke, blood product requirement, access-site bleeding, gastrointestinal bleeding, and other bleeding are per the NCDR CathPCI Registry data dictionary.

Ischemic MACE was defined as a composite of periprocedural myocardial infarction (MI) and stroke. PCI-related MI was defined by troponin elevation greater than 5 times the 99th percentile in patients with normal baseline values or a rise in troponin greater than 20% if baseline values were elevated. In addition, either symptoms suggestive of myocardial ischemia, new thrombotic electrocardiographic changes, angiographic findings consistent with a procedural complication, imaging evidence of loss of viable myocardium, or new regional wall motion abnormality were required.^21^ Stroke was defined as an acute episode of focal or global cerebral or spinal dysfunction caused by intraparenchymal, intraventricular, or subarachnoid hemorrhage or caused by brain, spinal cord, or retinal vascular injury as a result of infarction of central nervous system tissue.

The need for blood products was defined as packed blood cell transfusion between the start of the procedure and within 72 hours after the procedure or prior to discharge. Access-site bleeding was defined as observed and documented in the medical record associated with a hemoglobin drop greater than or equal to 3 g/dL or requiring blood transfusion. Other bleeding was defined as a confirmed bleeding event not available for selection within the registry that was observed and documented in the medical record associated with a hemoglobin drop greater than or equal to 3 g/dL, blood transfusion, or requiring procedural intervention to achieve hemostasis. Any bleeding complication was defined as a composite of all individual bleeding outcomes.

### Statistical methods

Categorical data are presented as percentages and continuous data are presented as mean ± SD. Independent samples *t* test, χ^2^, and Fisher exact tests were used as appropriate to compare patient characteristics, procedural characteristics, and complications between groups. Mortality was evaluated with time-to-event analysis using Kaplan-Meier curves with log-rank statistics to assess all-cause mortality at 30 days, 6 months, and 1 year. Wald χ^2^ testing was used to assess the goodness-of-fit. The Univariate Cox proportional hazards regression model was used to estimate hazard ratios for all-cause mortality. Adjusted mortality analysis using baseline patient demographics, clinical presentation, and procedural characteristics determined as confounders a priori was conducted with a multivariable Cox proportional hazards regression model. Variables used in the adjusted mortality analysis include age, sex, body mass index, smoking status, hypertension, hyperlipidemia, diabetes, dialysis dependence, ACS, prior MI, and prior PCI. Multivariable logistic regression with forward and backward procedures was used to determine odds ratios for independent predictors of RFC. Analyses were performed using IBM SPSS Statistics for MacOS, version 29 (IBM Corp); a 2-sided *P* < .05 was considered statistically significant.

## Results

### Patient characteristics and predictors of RFC

Of the 3054 patients included, 658 had FA, 2287 had RA, and 109 required RFC. The incidence of RFC among patients with attempted RA was 4.5%. Patient characteristics are shown in Table 1. The overall mean patient age was 67 ± 12 years, 28.9% female, 59.6% White race, and 23.9% Hispanic ethnicity. Hypertension (83.1%), hyperlipidemia (80.6%), congestive heart failure (41.2%), and diabetes (44.4%) were the most common comorbidities and 38.3% of patients had prior PCI. Patients who required RFC were older than patients who had RA. There were no differences in comorbidities between patients who required RFC and patients who had RA. Patients who had FA were more likely to have congestive heart failure and end-stage renal disease on dialysis compared to patients who required RFC. There were no differences in race or ethnicity between groups.

**Table 1:** Patient characteristics.

	Overall (N = 3054)	RFC (n = 109)	RA (n = 2287)	FA (n = 658)	*P* value RFC vs RA	*P* value RFC vs FA
Age, y	67 ± 12	71 ± 13	66 ± 12	69 ± 13	<.001	.218
Female sex	884 (28.9%)	33 (30.3%)	601 (26.3%)	250 (38.0%)	.456	.122
BMI, kg/m^2^	28.94 ± 6.92	28.61 ± 4.98	29.18 ± 6.74	28.16 ± 7.74	.386	.556
Smoker	1391 (45.5%)	53 (48.6%)	1037 (45.3%)	301 (45.7%)	.502	.577
Hypertension	2539 (83.1%)	94 (86.2%)	1873 (81.9%)	572 (86.9%)	.248	.843
Hyperlipidemia	2460 (80.6%)	90 (82.6%)	1796 (78.5%)	574 (87.2%)	.314	.186
CHF	1258 (41.2%)	43 (39.4%)	837 (36.6%)	378 (57.4%)	.546	<.001
Prior MI	787 (25.8%)	29 (26.6%)	544 (23.8%)	214 (32.5%)	.500	.219
Prior PCI	1171 (38.3%)	42 (38.5%)	835 (36.5%)	294 (44.7%)	.669	.231
ESRD on dialysis	184 (6.0%)	7 (6.4%)	69 (3.0%)	108 (16.4%)	.055	.007
Diabetes	1357 (44.4%)	48 (44.0%)	964 (42.2%)	345 (52.4%)	.697	.104
PAD	267 (8.7%)	10 (9.2%)	155 (6.8%)	102 (15.5%)	.334	.083
Lung disease	232 (7.6%)	12 (11.0%)	151 (6.6%)	69 (10.5%)	.074	.869
Race/ethnicity					.315	.341
Asian	54 (1.8%)	0 (0.0%)	34 (1.5%)	20 (3.0%)	–	–
Black	235 (7.7%)	10 (9.2%)	181 (7.9%)	44 (6.7%)	–	–
White	1821 (59.6%)	63 (57.8%)	1387 (60.6%)	371 (56.4%)	–	–
Hispanic	729 (23.9%)	32 (29.4%)	519 (22.7%)	178 (27.1%)	–	–

Values are mean ± SD for continuous variables and number and percentage of patients for categorical variables.

BMI, body mass index; CHF, congestive heart failure; ESRD, end-stage renal disease; FA, femoral access; MI, myocardial infarction; PAD, peripheral arterial disease; PCI, percutaneous coronary intervention; RA, radial access; RFC, radial to femoral access-site crossover.

Independent predictors of RFC were age greater than 70 years (OR, 2.68; 95% CI, 1.79-4.01; *P* < .001), vasopressor support at the time of PCI (OR, 2.87; 95% CI, 1.33-6.20; *P* = .007), and dialysis dependence (OR, 3.05; 95% CI, 1.34-6.93; *P* = .008).

### Reasons for RFC and procedural characteristics

Reasons for RFC are shown in the Central Illustration (A). In 34.9% of cases, RFC was required before sheath insertion for failed radial puncture (19.3%) or inability to advance the wire or sheath (15.6%). Operators attempted ultrasound-guided RA in 31.2% of these cases before switching to FA. Ultrasound-guided RA was not used upfront in any of the RFC cases. In 65.1% of cases, RFC was required after sheath insertion for tortuosity (31.2%), vasospasm (22.0%), inability to engage (5.5%), poor guide support (2.8%), inability to cross the lesion (1.8%), or inability to deploy a stent (1.8%).

**Central Illustration:**
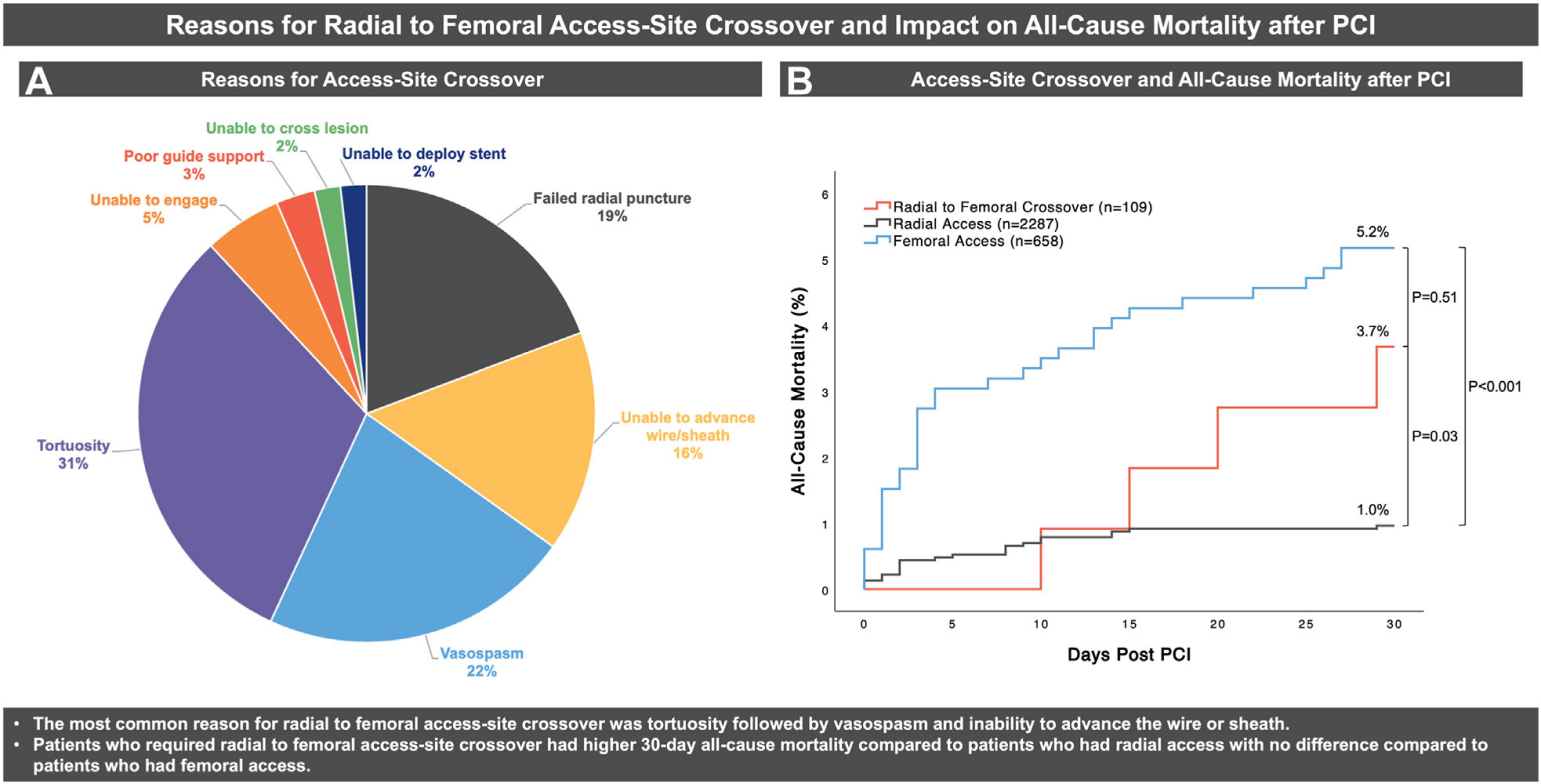
(A) Pie chart of reasons for radial to femoral access-site crossover. (B) Kaplan-Meier survival estimates for all-cause mortality at 30 days post-PCI by access site. PCI, percutaneous coronary intervention.

Procedural characteristics are shown in Table 2. Patients presented with STEMI (12.2%), non-STEMI (31.0%), unstable angina (1.2%), stable angina (38.0%), and most patients had non-left main one vessel disease. The FA cohort had a greater proportion of patients with stable angina whereas the RFC and RA cohorts had a greater proportion of patients with ACS.

**Table 2:** Procedural characteristics.

	Overall (N = 3054)	RFC (n = 109)	RA (n = 2287)	FA (n = 658)	*P* value RFC vs RA	*P* value RFC vs FA
Presentation					.686	.014
STEMI	374 (12.2%)	16 (14.7%)	321 (14.0%)	37 (5.6%)	–	–
NSTEMI	946 (31.0%)	34 (31.2%)	726 (31.7%)	186 (28.3%)	–	–
Unstable angina	36 (1.2%)	0 (0.0%)	30 (1.3%)	6 (0.9%)	–	–
Stable angina	1163 (38.0%)	37 (33.9%)	818 (35.6%)	308 (46.4%)	–	–
Other PCI indication	516 (16.9%)	22 (20.2%)	382 (16.7%)	112 (17.0%)	–	–
Lesion findings					.431	.048
1VD not LM	2480 (81.2%)	98 (89.9%)	1891 (82.7%)	491 (74.6%)	–	–
2VD not LM	334 (10.9%)	7 (6.4%)	240 (10.5%)	87 (13.2%)	–	–
3VD not LM	42 (1.4%)	2 (1.8%)	27 (1.2%)	13 (2.0%)	–	–
LM + 1VD	36 (1.2%)	0 (0.0%)	12 (0.5%)	24 (3.6%)	–	–
LM + 2VD	17 (0.6%)	0 (0.0%)	8 (0.3%)	9 (1.4%)	–	–
LM + 3VD	2 (0.1%)	0 (0.0%)	1 (0.0%)	1 (0.2%)	–	–
LM	28 (0.9%)	0 (0.0%)	13 (0.6%)	15 (2.3%)	–	–
Fluoroscopy time, min	23 ± 16	27 ± 16	21 ± 12	32 ± 24	<.001	.036
Contrast volume, mL	157 ± 80	170 ± 73	147 ± 69	190 ± 106	.001	.055
STEMI door-to-reperfusion time, min	68 ± 32	79 ± 45	67 ± 32	66 ± 17	.299	.489
Days of admission	3 ± 6	4 ± 7	3 ± 5	5 ± 8	.014	.416
Periprocedural medication						
Heparin	2722 (89.1%)	93 (85.3%)	2155 (94.2%)	474 (72.0%)	<.001	.003
Bivalirudin	1350 (44.2%)	69 (63.3%)	971 (42.5%)	310 (47.1%)	<.001	.002
GPI	46 (1.5%)	1 (0.9%)	39 (1.7%)	6 (0.9%)	.449	1.000
Cangrelor	532 (17.4%)	24 (22.0%)	413 (18.1%)	95 (14.4%)	.296	.043
Aspirin	2930 (95.9%)	105 (96.3%)	2189 (95.7%)	636 (96.7%)	.500	1.000
Clopidogrel	1761 (57.7%)	67 (61.5%)	1265 (55.3%)	429 (65.2%)	.206	.451
Ticagrelor	1128 (36.9%)	35 (32.1%)	898 (39.3%)	195 (29.6%)	.134	.601
Prasugrel	43 (1.4%)	0 (0.0%)	34 (1.5%)	9 (1.4%)	.203	.373

Values are mean ± SD for continuous variables and number and percentage of patients for categorical variables.

FA, femoral access; GPI, glycoprotein IIb/IIIa inhibitors; LM, left main; NSTEMI, non–ST-segment elevation myocardial infarction; RA, radial access; RFC, radial to femoral access-site crossover; STEMI, ST-segment elevation myocardial infarction; VD, vessel disease.

Patients who required RFC had longer fluoroscopy time (27 ± 16 minutes vs 21 ± 12 minutes; *P* < .001) and received more contrast volume (170 ± 73 mL vs 147 ± 69 mL; *P* = .001) compared to patients who had RA. In STEMI, patients who required RFC had a longer door-to-reperfusion time compared to patients who had RA (79 ± 45 minutes vs 67 ± 32 minutes; *P* = .299) or FA (79 ± 45 minutes vs 66 ± 17 minutes; *P* = .489) but this did not reach statistical significance. Patients who required RFC had longer admission duration (4 ± 7 days vs 3 ± 5 days; *P* = 0.014) compared to patients who had RA.

Periprocedural medications administered included heparin (89.1%), bivalirudin (44.2%), cangrelor (17.4%), aspirin (95.9%), clopidogrel (57.7%), and ticagrelor (36.9%). Few patients received glycoprotein IIb IIIa inhibitors (1.5%) or prasugrel (1.4%). Patients who required RFC were more likely to receive bivalirudin compared to patients who had RA or FA. Patients who required RFC were more likely to receive cangrelor compared to patients who had FA.

### Bleeding complications and ischemic MACE

Of the 3054 patients, 133 patients (4.4%) had a bleeding complication with 40 patients (1.3%) having an access-site bleed, 24 patients (0.8%) with a gastrointestinal bleed, 4 patients (0.1%) with a genitourinary bleed, and 65 patients (2.1%) with another bleed. Bleeding Academic Research Consortium (BARC) 3 or above bleeding occurred in 98 patients (3.2%) and blood product transfusion was required in 98 (3.2%) patients. Ischemic MACE occurred in 21 patients (0.7%).

Patients who had FA had a higher rate of bleeding complications (9.9% vs 2.6%; *P* < .001), access-site bleeding (3.2% vs 0.7%; *P* < .001), BARC 3 or above bleeding (8.8% vs 1.4%; *P* < .001), and blood product transfusion (8.8% vs 1.4%; *P* < .001) compared to patients who had RA with no difference in ischemic MACE.

Among patients who required RFC, 9 patients (8.3%) had a bleeding complication, 2 patients (1.8%) had bleeding at the percutaneous entry site, 3 patients (2.8%) had gastrointestinal bleeding, and 4 patients (3.7%) had another bleed. There were no ischemic MACE events among patients who required RFC. Patients who required RFC had a higher rate of bleeding complications (8.3% vs 2.6%; *P* = .003), BARC 3 or above bleeding (7.3% vs 1.4%; *P* < .001), and blood product transfusion (7.3% vs 1.4%; *P* < .001) compared to patients who had RA. There was no difference in ischemic MACE between patients who required RFC and patients who had RA. There was no difference in bleeding complications or ischemic MACE between patients who required RFC and patients who had FA.

### Unadjusted and adjusted all-cause mortality post-PCI

In the entire population, there was 2.0% 30-day all-cause mortality, 4.1% 6-month all-cause mortality, and 6.1% 1-year all-cause mortality (Table 3). Patients who had FA had higher all-cause mortality at 30 days (5.2% vs 1.0%; *P* < .001), 6 months (9.7% vs 2.4%; *P* < .001), and 1 year (13.2% vs 4.0%; *P* < .001) compared to patients who had RA. Patients who required RFC had higher 30-day all-cause mortality compared to patients who had RA (3.7% vs 1%; *P* = .028) (Central Illustration, B) with no difference in 6-month or 1-year all-cause mortality. The hazard ratio for 30-day all-cause mortality after PCI was 3.81 (95% CI, 1.31-11.06; *P* = .014) for RFC compared to RA. In the adjusted mortality analysis, patients who required RFC also had significantly higher all-cause mortality at 30 days (HR, 3.40; 95% CI, 1.14-10.14; *P* = .028) compared to patients who had RA. There was no difference in mortality at any time point between patients who required RFC and patients who had FA. Patients who had bleeding complications, irrespective of access-site, had higher all-cause mortality at 30 days (17.3% vs 1.3%; *P* < .001), 6 months (24.8% vs 3.2%; *P* < .001), and 1 year (28.6% vs 5.1%; *P* < .001) compared to patients who did not have bleeding complications.

**Table 3:** Procedural complications and all-cause mortality.

	Overall (N = 3054)	RFC (n = 109)	RA (n = 2287)	FA (n = 658)	*P* value RFC vs RA	*P* value RFC vs FA	*P* value RA vs FA
Any bleeding complication	133 (4.4%)	9 (8.3%)	59 (2.6%)	65 (9.9%)	.003	.595	<.001
Access-site bleeding	40 (1.3%)	2 (1.8%)	17 (0.7%)	21 (3.2%)	.213	.560	<.001
Gastrointestinal bleeding	24 (0.8%)	3 (2.8%)	14 (0.6%)	7 (1.1%)	.039	.159	.289
Genitourinary bleeding	4 (0.1%)	0 (0.0%)	1 (0.0%)	3 (0.5%)	1.000	1.000	.037
Other bleeding	65 (2.1%)	4 (3.7%)	27 (1.2%)	34 (5.2%)	.049	.505	<.001
BARC 3 bleeding	98 (3.2%)	8 (7.3%)	32 (1.4%)	58 (8.8%)	<.001	.611	<.001
Need for blood products	98 (3.2%)	8 (7.3%)	32 (1.4%)	58 (8.8%)	<.001	.611	<.001
Any ischemic MACE	21 (0.7%)	0 (0.0%)	18 (0.8%)	3 (0.5%)	.627	1.000	.445
Periprocedural MI	11 (0.4%)	0 (0.0%)	10 (0.4%)	1 (0.2%)	1.000	1.000	.474
CVA	10 (0.3%)	0 (0.0%)	8 (0.3%)	2 (0.3%)	1.000	1.000	1.000
30-day mortality	60 (2.0%)	4 (3.7%)	22 (1.0%)	34 (5.2%)	.028	.505	<.001
6-month mortality	126 (4.1%)	6 (5.5%)	56 (2.4%)	64 (9.7%)	.060	.156	<.001
1-year mortality	187 (6.1%)	8 (7.3%)	92 (4.0%)	87 (13.2%)	.133	.084	<.001

Values are the number and percentage of patients.

BARC, Bleeding Academic Research Consortium; CVA, cerebrovascular accident; FA, femoral access; MACE, major adverse cardiovascular event; MI, myocardial infarction; RA, radial access; RFC, radial to femoral access-site crossover.

## Discussion

In a large cohort of patients who underwent PCI for any indication with long-term follow-up, we found that RFC was associated with a loss of the bleeding and mortality benefit of RA without added risk compared to FA. We evaluated the impact of RFC on post-PCI mortality in both ACS and non-ACS in the modern era with widespread operator familiarity with RA and ultrasound guidance.

We observed RFC crossover rates of 4.5% even in a high-volume radial center which is on the lower end of previously published rates. One-fifth of RFC cases were due to failed radial puncture and notably ultrasound was not used upfront in any of these cases. Ultrasound guidance was employed in some of these cases after failed radial puncture but was still unsuccessful requiring a bailout to FA. This may be a result of radial artery compromise due to failed attempts resulting in vasospasm or vessel injury. Initial ultrasound-guided RA may have mitigated RFC in these cases as it has been shown to improve the success rates and efficiency of RA.^22^

Two-thirds of RFC cases occurred after radial sheath insertion, most commonly for tortuosity followed by vasospasm. If resistance is encountered while advancing wires or catheters, the vessel anatomy should be defined by injecting from the sheath. Tortuous vessels can be navigated by using hydrophilic guide wires, standard coronary wires, or balloon-assisted tracking techniques.^23^^,^^24^ Left RA has been associated with significantly lower operator-reported tortuosity compared to right RA.^25^ Guidelines recommend considering an a priori left radial approach in certain patient populations including the elderly.^26^ Distal radial and ulnar access also have comparable efficacy to RA.^27^, ^28^, ^29^ Given the known benefits of forearm arterial access, perhaps bailout to forearm arterial access first rather than FA could have been attempted in these cases. The various mechanisms to prevent and mitigate vasospasm should also be emphasized including adequate sedation and analgesia, administration of intra-arterial vasodilators, and use of small-caliber sheaths.^7^ Ultrasound guidance can facilitate arterial access with fewer attempts thereby also minimizing vasospasm.

Older age was associated with RFC as reported previously.^11^^,^^16^^,^^17^^,^^19^^,^^30^ There was a greater proportion of patients with end-stage renal disease on dialysis in the RFC group compared to the RA group though this did not reach statistical significance. Unlike previous studies, we found no difference in sex, body size, or other comorbidities between patients who required RFC compared to patients who had RA. We identified age over 70 years, vasopressor use at the time of PCI, and dialysis dependence as predictors of RFC. These patients are also at inherently higher risk of procedural complications and mortality with FA. This subgroup of patients may benefit from initial ultrasound-guided RA and bailout to left RA before FA, especially in non-ACS settings.

Patients who required RFC received more contrast volume, had longer fluoroscopy times, and had longer admission duration compared to patients who had RA but not compared to patients who had FA. In STEMI, there was a higher door-to-reperfusion time with RFC although this did not reach statistical significance because we were underpowered to detect differences in subgroups. Certain STEMI patients may benefit from initial ultrasound-guided RA or FA. This needs to be elucidated further in future studies.

RFC was associated with a higher rate of major bleeding complications and a higher need for blood products compared to RA. These bleeding events were likely procedure-related and drove the observed higher 30-day mortality rate with RFC compared to RA which persisted in our adjusted analysis. There were no differences in long-term post-PCI mortality between RFC and RA. Reassuringly, there were no differences in outcomes between RFC and FA despite the presence of multiple access sites. We also observed no difference in ischemic MACE regardless of access route.

Despite the routine use of ultrasound guidance for FA and closure devices, major bleeding complications including access-site bleeding and post-PCI mortality remain higher with FA compared to RA. Thus, a radial first approach should be employed. Failed RA requiring crossover to FA is an enduring procedural challenge associated with greater major bleeding complications and higher short-term mortality compared to RA. Universal ultrasound guidance for RA has consistently demonstrated improved efficiency and success rates, regardless of patient characteristics. We identify patients at high risk for RFC who may benefit from initial ultrasound-guided RA to maximize the chances of successful RA. When right RA fails, patient outcomes may be improved by crossover to left RA before FA in certain situations.

### Limitations

The limitations of this study include its retrospective nature using registry data. Only radial to femoral crossover events and bleeding complications were adjudicated. This was a single-center study conducted from PCI procedures that occurred at a high-volume academic medical center in the Unites States with operator expertise in RA so results may not be generalizable to all institutions. Access-site–related management decisions were at the discretion of the primary operator which introduces variability but is representative of clinical practice.

## Conclusion

In this study, we found that RFC was associated with higher 30-day all-cause mortality post-PCI and major bleeding complications compared to RA. Age greater than 70 years, vasopressor support, and dialysis dependence were associated with RFC.
